# The Combined Thermoresponsive Cell-Imprinted Substrate, Induced Differentiation, and "KLC Sheet" Formation

**DOI:** 10.34172/apb.2022.034

**Published:** 2021-05-02

**Authors:** Neda Keyhanvar, Nosratollah Zarghami, Alexander Seifalian, Peyman Keyhanvar, Rana Sarvari, Roya Salehi, Reza Rahbarghazi, Mohammadreza Ranjkesh, Molood Akbarzadeh, Mahdi Mahdipour, Mohammad Nouri

**Affiliations:** ^1^Stem Cell Research Center, Tabriz University of Medical Sciences, Tabriz, Iran.; ^2^Department of Medical Biotechnology, Faculty of Advanced Medical Sciences, Tabriz University of Medical Sciences, Tabriz, Iran.; ^3^Stem Cell and Regenerative Medicine Institute, Tabriz University of Medical Sciences, Tabriz Iran.; ^4^Nanotechnology and Regenerative Medicine Centre (Ltd), the London BioScience Innovation Centre, London, UK.; ^5^Department of Medical Nanotechnology, Faculty of Advanced Medical Sciences, Tabriz University of Medical Sciences, Tabriz, Iran.; ^6^Infectious and Tropical Diseases Research Center, Tabriz University of Medical Sciences, Tabriz, Iran.; ^7^Department of Applied Cell Sciences, Faculty of Advanced Medical Sciences, Tabriz University of Medical Sciences, Tabriz, Iran.; ^8^Dermatology & Dermopharmacy Research Team and Department of Dermatology, Sina Hospital, Tabriz University of Medical Sciences, Tabriz, Iran.; ^9^Department of Cellular and Molecular Biology, Faculty of Biological Science, Azarbaijan Shahid Madani University, Tabriz, Iran.; ^10^Department of Reproductive Biology, Faculty of Advanced Medical Sciences, Tabriz University of Medical Sciences, Tabriz, Iran.

**Keywords:** Adipose tissue-derived mesenchymal stem cells, Cell-imprinting, Cell sheet engineering, Differentiation, Poly N-Isopropyl acrylamide (PNIPAAm), Topography

## Abstract

***Purpose:*** Stem cells can exhibit restorative effects with the commitment to functional cells.Cell-imprinted topographies provide adaptable templates and certain dimensions for thedifferentiation and bioactivity of stem cells. Cell sheet technology using the thermo-responsivepolymers detaches the "cell sheets" easier with less destructive effects on the extracellularmatrix (ECM). Here, we aim to dictate keratinocyte-like differentiation of mesenchymal stemcells (MSCs) by using combined cell imprinting and sheet technology.

***Methods:*** We developed the poly dimethyl siloxane (PDMS) substrate having keratinocytecell-imprinted topography grafted with the PNIPAAm polymer. Adipose tissue-derived MSCs(AT-MSCs) were cultured on PDMS substrate for 14 days and keratinocyte-like differentiationmonitored via the expression of involucrin, P63, and cytokeratin 14.

***Results:*** Data showed the efficiency of the current protocol in the fabrication of PDMSmolds. The culture of AT-MSCs induced typical keratinocyte morphology and up-regulatedthe expression of cytokeratin-14, Involucrin, and P63 compared to AT-MSCs cultured on theplastic surface (*P* < 0.05). Besides, KLC sheets were generated once slight changes occur in theenvironment temperature.

***Conclusion:*** These data showed the hypothesis that keratinocyte cell imprinted substrate canorient AT-MSCs toward KLCs by providing a specific niche and topography.

## Introduction

Skin is known as the body`s largest organ which plays an important role as a barrier to the external environment. As a correlate, cutaneous tissue regeneration is vital once different pathologies occur.^1-3^ This organ can restore the injured site via provoking resident stem/progenitor cells.^2^ These cells are primarily unspecialized with prominent self-renewal and differentiation into multiple lineages.^4^ In normal cutaneous tissue, the basal layer of the adult epidermis harbor distinct stem cells namely basal cells with the ability to mature to functional keratinocytes.^5^ However, during different cutaneous injuries, in most areas of the skin is lost, and thus the healing procedure postpone. Therefore, any attempts have been focused to use alternative stem cell sources for the regeneration of injured skin. Among all cell/stem cell types, mesenchymal stem cells (MSCs), especially adipose-derived MSCs (AT-MSCs), are the most widely used cells in the transplantation into the injured tissues.^6^ AT-MSCs are easily isolated from adult donors without invasive surgical approaches. Besides, the existence of inherent immunomodulatory properties makes these cells an efficient candidate in regenerative medicine.^7^

Cell sheet engineering allows the transplantation of confluent cell layer to the injured surfaces using thermoresponsive smart biomaterials.^8^ In this method, which was first reported by Yamato and Okano, cells are expanded on ready-to-use culture dishes (UpCell^®^) or culture plates coated with certain thermoresponsive polymers like PNIPAAm.^9^ Thus, environmental factors such as surrounding temperature are vital in the control of each cell’s behavior.^10^

Like specific substrates and scaffolds, growth factors possess a significant role in stem cell differentiation.^11^ It has been shown that the combination of both scaffold and growth factors can facilitate the orientation of stem cells toward target lineages. Particular physicochemical properties of ECM dictate specific cell responses by engaging relevant signaling pathways.^12-18^ In addition, the topographical feature of each scaffold should not be neglected in which in *in vivo* milieu cells function are tightly regulated by the ECM components and topographical indices. Cells might face several topographical patterns including macro, micro, and nanoscale features during maturation and dynamics growths. During the expansion of cells in in vitro conditions, most of these clues are lacking.^19-22^ In response to topographical patterns such as dots, pores, columns, meshwork, pits, gratings, and random surface shapes, cells change adhesion, morphology, proliferation, cytoskeletal formation, gene expression, migration, and even surface antigens.^10,12,13,22-24^ For example, Unadkat et al. created 2176 different surface topographies with different sizes, heights, and shapes.^25^ In another study conducted by Markert et al, they used 504 different topographies to promote differentiation of embryonic stem cells. They showed that these topographies can be used instead of feeder cells.^26^

Despite the importance of surface topography in stem cell biology, the most used cell culture plates and flasks are still made of rigid and non-patterned materials. Knowing this, cell-imprinting is reverse engineering of the cell surface patterns for cell culture approaches.^27^ Along with all the techniques used to develop topographical surfaces, direct molding of the cell shapes (cell imprinting) is known to be a more efficient method to affect stem cells morphological alignment, elongation, polarization, migration, proliferation, and gene expression toward desired differentiation status.^10,27^ For example, in 2014, Lee et al developed myoblast imprinted substrates for the culture of MSCs.^28^ They confirmed appropriate morphological adaptation and myogenic differentiation of MSCs on the myometrium-like biomimetic substrate.^28^ The number of studies related to stem cell culture on cell-imprinted patterns is increasing time by time.^29-39^ Here, we aimed to examine whether the culture of AT-MSCs on keratinocyte cell imprinted substrate and cell sheet engineering technology can dictate differentiation toward KLC.

## Materials and Methods

### AT-MSCs culture and expansion

**Figure 1:**
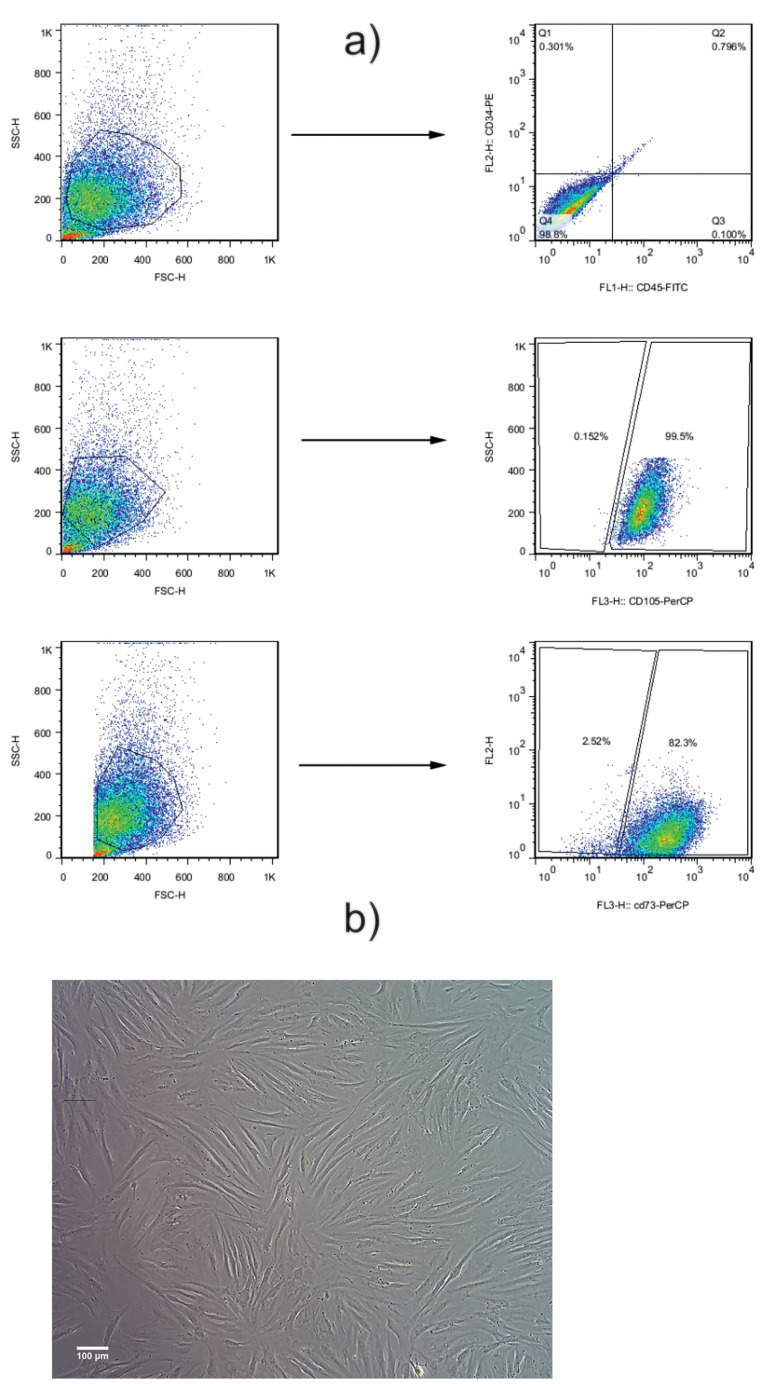
Flow cytometry analysis of the AT-MSCs. Results indicated that: 1b) more than 99% was negative for CD34, and 1c) more than 98% was negative for CD45. 1d) Around 97% were positive for CD73 and 1e) more than 99% were positive for CD105. These cells were used for cell culture and further experiments. 1f) AT-MSCs exhibited a spindle fibroblast-like shape, having the capacity to attach and proliferate in desired culture conditions. All images in this figure were captured with the MICROS Sundew MCXI600 Inverted Biological Microscope.

Human AT-MSCs were provided by the Azerbaijan Stem Cell and Regenerative Medicine Institute (SCARM) at the Tabriz University of Medical Sciences. Cells were previously characterized by flow cytometry analysis. Cells were cultured in Dulbecco’s Modified Eagle’s Medium/Ham’s F12 (DMEM/F12) (Gibco, Scotland) supplemented with 10% (v/v) fetal bovine serum (FBS) (Seromed, Germany), 100 IU/ml penicillin, and 100 µg/ml streptomycin (Sigma, USA). Cells were maintained in a humidified atmosphere at 37°C with 5% CO2. The medium was changed every 3-4 days until 70-80% confluency. AT-MSCs were detached using TrypLE^TM^ (Gibco, UK). Cells at passages 3 to 4 were subjected to different analyses. Characterization of the AT-MSCs is summarized in Figure 1.

#### Keratinocytes isolation from neonatal foreskin

#### Sample preparation and epidermal isolation

To this end, parents were asked to complete informed consent before sampling. Keratinocytes were isolated from foreskin samples of newborn infants during circumcision. Briefly, samples were collected in 50 mL falcons containing 5 ml Hank’s Balanced Salt Solution (HBSS) (Gibco, UK) enriched with 7.5 mg/mL fungizone (Gibco, UK), 300 U/mL penicillin, and 300 mg/mL streptomycin (Gibco, UK). The samples were transferred to the cell culture laboratory. Before isolation, samples were disinfected in 70% EtOH for 40 seconds and washed three times with phosphate-buffered saline (PBS) (Gibco, UK). Thereafter, subcutaneous fat and connective tissues were carefully removed and samples were cut into small pieces (0.5 cm × 0.5 cm). For enzymatic digestion, samples were incubated with 2.5 mg/mL dispase in HBSS (Gibco, UK) at 4°C overnight (16-18 hours). The next day, samples were transferred to a sterile petri dish and the epidermis was separated from the dermis using forceps.

#### Trypsinization of epidermis layer

Epidermal tissue in PBS was cut into very small pieces (2 mm × 2 mm) and transferred to a 15 mL Falcone tube having the appropriate amount of TrypLE^TM^ (Gibco**)** enzyme for 15-20 minutes and the tube was shaken every 3 minutes for better exposure and digestion. Finally, the solution was pipetted up and down (25-30 times) and passed through a 70 µm mesh filter to eliminate undigested tissue and transferred to another sterile tube. Cells were centrifuged at 200×g for 5 minutes and the cell pellet was re-suspended in keratinocyte specific medium (Epilife medium) supplemented with human keratinocyte growth factor supplemented with 100 U/mL penicillin and 100 mg/mL streptomycin and cultured in a 25 cm culture flask. To achieve the optimum result,type I collagen as a Coating Matrix was used to coat the culture plates. Cells were incubated at 37°C, 5% CO_2,_ and 95% humidity. The medium was changed every other day until confluency. Cells were detached using the TrypLE^TM^ enzyme for follow-up experiments.

#### Fabrication of cell-imprinted substrate

For this purpose, poly dimethyl siloxane (PDMS) (SYLGARD^®^ 184, RTV, Dow Corning, USA) was used to fabricate the cell-imprinted substrates. Briefly, keratinocytes were cultured in EpiLife^®^ basal medium supplemented with Keratinocyte Medium Supplement. Upon reaching 70-80% confluence, the supernatant culture medium was discarded. Then, the PDMS substrate was fixed in 4% glutaraldehyde and washed with PBS several times. To molding, we mixed silicone resin and curing agent at a ratio of 10: 1 according to the manufacturer’s instruction. The mixture was degassed by vacuum, and heated at 45°C for 30-35 minutes. After cooling, the cured silicone was poured onto the wells containing fixed cell samples and incubated at 37°C for 24-48 hours for obtaining the imprinted substrates. Thereafter, cured silicone was peeled off from cell culture plates and the imprinted surfaces were washed with boiling water and 1 M NaOH solution to remove the cell debris. The cell-imprinted substrate was observed by a scanning electron microscope (SEM) microscopy.

#### Thermoresponsive substrate development

#### Ultraviolet/Ozone (UV/O) treatment

The ultraviolet/ozone (UV/O) treatment of the PDMS surface was done in a commercial UV/O chamber (Jelight Company, Inc., Model 42-220, Irvine, CA). This method is a kind of an oxidation process in which surface molecules are excited exposed to the short-wavelength UV radiation. The atomic oxygen and ozone are generated by λ1 = 184.9 nm and by λ2 = 253.7 nm, respectively. The 253.7 nm radiation can be absorbed by most of the hydrocarbons. Therefore, in the presence of wavelengths, atomic oxygen and ozone are continuously generated. The Sylgard-184 PDMS substrate was placed into the UV/O cleaner tray at a 6 mm distance from the UV source and exposed to the radiation for 20 minutes.

#### Contact angle analysis

The hydrophobicity of PDMS can limit the successful culture of certain cell types.^40^ The UV/O treatment was performed to make the PDMS surface more hydrophilic for NIPAAm grafting. Following the surface modification, surface wettability will be improved, but the PDMS polymer chains will rearrange which is called “hydrophobic recovery”.^40^ Contact angle goniometry was performed using a home-built contact angle measurement device equipped with a TZM-2 microscope (BEL engineering company, Italy) coupled to a 3 megapixel CMOS digital camera. The reported contact angle values corresponded to the mean of three independent measurements. The advancing contact angles were read within 30 s after treatment and the receding contact angles were determined by removing 4 µL from the deionized water (DIW) droplet. As detailed later, these contact angle data were used to estimate the surface energy of the solid.

#### PNIPAAm grafting on treated PDMS surface

For this aim, two different methods including UV and atom transfer radical polymerization (ATRP) were applied to polymerize PNIPAAm on the functionalized PDMS surface.

UV polymerization method: UV/O treated PDMS substrates were immediately immersed in the N-isopropyl acrylamide (NIPAAm) monomer solution (20% w/v in 2-propanol) and placed in the UV chamber for both 15 and 60 minutes. The distance between the UV lamp and the substrate was adjusted to 10 mm.

ATRP polymerization method: For this aim, the initiator was prepared by the reaction of (3-aminopropyl) trimethoxysilane with BIBB in the presence of the triethylamine. In the next step, UV/O-treated PDMS substrate was immersed in an initiator solution (0.3 g/cm^2^) in dry ethanol (35 mL) at room temperature for 24 hours under an argon atmosphere. Thereafter, the composite was washed with ethanol to remove residual initiators followed by drying under vacuum. Grafting of PNIPAAm on macroinitiator surface was carried out via ATRP with a reaction system containing NIPAAm/bipyridine/CuCl at a molar ratio of 500/20/10 in methanol. In a 100 ml flask, the macroinitiator was immersed in a solvent (20 mL) and sonicated. The system was degassed by argon purging for 15 minutes. Then, CuCl and bipyridine, and NIPAAm were added respectively. The mixture was stirred under argon flow for 3 hours and placed at 50°C in an oil bath. After 24 hours, the polymerization was stopped and the product washed with methanol to remove the residue monomer and homopolymers from the surface. The polymer was dried under vacuum for 48 hours.

PNIPAAm grafting evaluation: Fourier transform infrared spectroscopy in the attenuated total reflection mode (FTIR-ATR) was used for characterizing chemical changes on the surface of the PDMS substrate after UV and ATRP polymerization methods. The spectra were recorded using a Tensor 27 (Bruker, Germany) spectrometer equipped with an attenuated total reflectance (ATR) accessory at 600 scans with a 4 cm-1 resolution.

#### Human AT-MSCs culture on a developed substrate

The fabricated substrates were immersed in 70% ethanol for 1 hour (Merck, Germany), cut to the diameter of a well in the 12-well plates. Before cell culture, the imprinted surface inside the wells was exposed to the UV for 40 minutes. An initial number of 3×10^4^ cells/cm^2^ AT-MSCs in 200 µL DMEM/F12 culture medium were poured onto the cell-imprinted substrates. The next day, 800 µL fresh medium supplemented with 10% (v/v) FBS was added to each substrate. Keratinocyte cells and AT-MSCs were cultured on the plastic surface to compare the differences between groups. In all the groups, cells were cultured for 14 days and the medium was changed every 3-4 days.

#### Quantitative real-time PCR

On day 14, total RNA content was extracted from all groups using the RiboEx kit (GeneAll^®^, Seoul, Korea) according to the manufacture’s instruction. The concentration of RNA was quantified at the wavelength of 260 nm using a spectrophotometer instrument (Thermo Scientific^TM^ NanoDrop^TM^ One, USA). RNA samples were reverse-transcribed into cDNA using the HyperScript^TM^ (GeneAll^®^, Seoul, Korea). Expression of keratinocyte-specific markers such as Cytokeratin 14 (K14), Involucrin (Inv), and P63 was monitored (Table 1). All reactions were carried out in triplicate. β-actin was used as an internal control gene. 2^-∆∆CT^ was used to calculate the relative expression of target genes.

**Table 1:** Primer sequence pairs used in qPCR.

**Spices**	**Name**	**Forward sequence**	**Reverse sequence**
Human	Cytokeratin 14(K14)	GGCCTGCTGAGATCAAAGAC	GGTTCAACTCTGTCTCATACTTGG
Human	Involucrin (Inv)	CTCTGCCTCAGCCTTACTG	CAGTGGAGTTGGCTGTTTCA
Human	P63	TCAACGAGGGACAGATTGCC	CAACCTGGGGTGGCTCATAA
Human	Β-actin	CAAGATCATCACCAATGCCT	CCCATCACGCCACAGTTTCC

### Cell sheet detachment analysis

To affirm the thermosensitive properties, AT-MSCs were cultured on cell-imprinted PDMS substrate and kept for 14 days. After reaching appropriate confluency, the normal culture medium was replaced with a pre-chilled (4°C) medium.

### Histological evaluation

The developed cell sheet with AT-MSCs was fixed in 10% formalin solution, embedded in paraffin blocks and 5 µm-thick sections prepared. Slides were stained with hematoxylin and eosin (H&E) solution.

#### Statistical Analysis

All data were expressed as means ± SD of three independent experiments and analyzed with one-way ANOVA and pair-wise multiple comparison procedures (Tukey tests) using GraphPad PRISM software ver. (8.0.1). *P* values < 0.05 were considered statistically significant.

## Results and Discussion

### Adipose tissue-derived mesenchymal stem cell isolation and expansion

Flow cytometry analysis indicated that more than 99% were positive for CD105 (Figure 1a). According to our data, near 82% were positive for CD73. Also, they were more than 98% was double negative for hematopoietic cell markers CD45 and CD34. These data showed MSC-like phenotype in the cultured cells. Bright-field imaging showed a typical spindle fibroblast-like shape (Figure 1b).

### Primary human keratinocyte isolation

**Figure 2:**
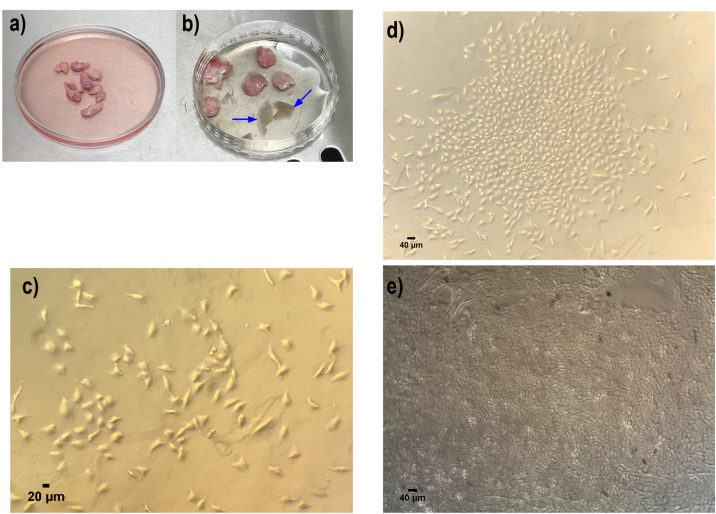
Primary cell isolation and culture of human keratinocyte from neonatal foreskin samples. (a) Neonatal foreskin samples in DMEM medium containing 1% Penicillin/streptomycin. (b) Isolated epidermis (illustrated with blue arrow) from dermis for further pure keratinocyte isolation. (c) Primary human keratinocyte culture in day 3 (d) day 7 and (e) at passage 1. All these indicated the well attachment and proliferation of the keratinocyte cells, which shows the polygonal phenotype. All images in this figure were captured with the MICROS Sundew MCXI600 Inverted Biological Microscope.

As shown in Figure 2, data revealed successful isolation of primary keratinocytes. The morphology of expanded cells is typical and similar to uniform epidermis keratinocytes (Figure 2).

### Cell-imprinted thermoresponsive PDMS substrate analysis

#### SEM image analysis

**Figure 3:**
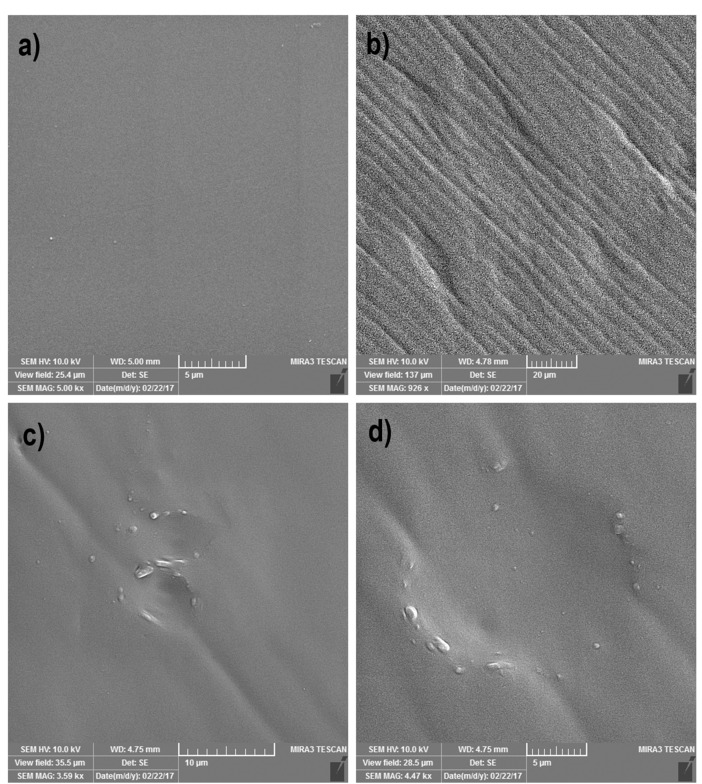
SEM image analysis of the negatively keratinocyte cell-imprinted PDMS substrate. (a) is the representative for the plane control PDMS substrate without imprinted groove. (b-d) Shows the formation of the successful grooves similar to the keratinocyte cell shape and morphology with an about 17.05 µm diameter.

According to SEM imaging, cell imprinted PDMS substrate was successfully developed. Data showed the successful formation of cell shape grooves on PDMS silicon which are comparable to the keratinocytes shape (Figure 3).

#### UV/O treatment and contact angle analysis

**Figure 4:**
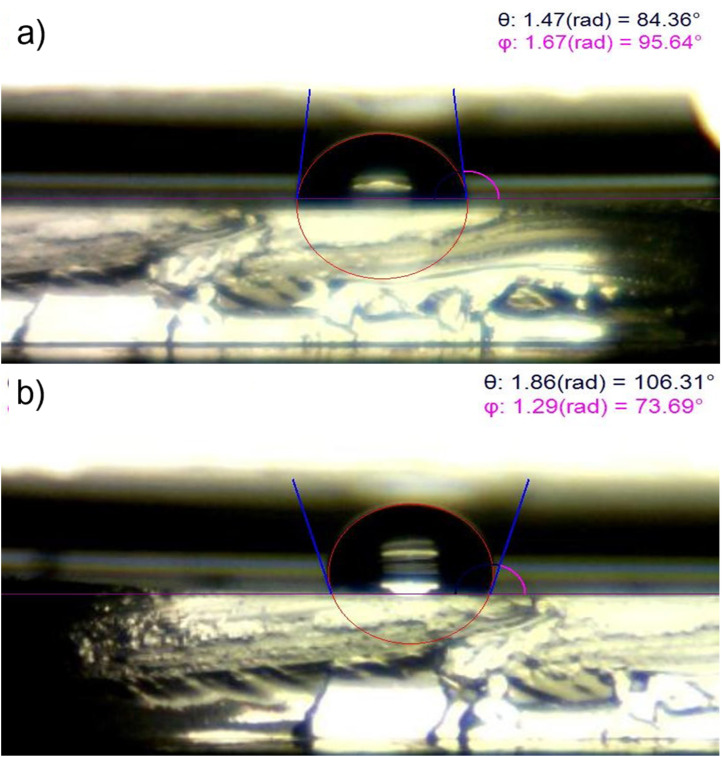
Contact angle measurement of the untreated and UV/O treated PDMS. UV/O treatment of the PDMS surface decreased the contact angle to 84.36° and makes it more hydrophilic (a) compared to the Untreated PDMS surface which is hydrophobic with contact angle about 106.31° (b).

We noted that the PDMS surface is hydrophobic, with a contact angle of 106.0° and these values reached 84.0° following the addition of hydroxyl (-OH) or silanol (-SH) groups after treatment with UV/O (Figure 4). Commensurate with these comments, –OH or -SH groups were generated on the PDMS surface to attach to the NIPAAm monomer under UV irradiation and ATRP method.

#### ATR-FTIR analysis

**Figure 5:**
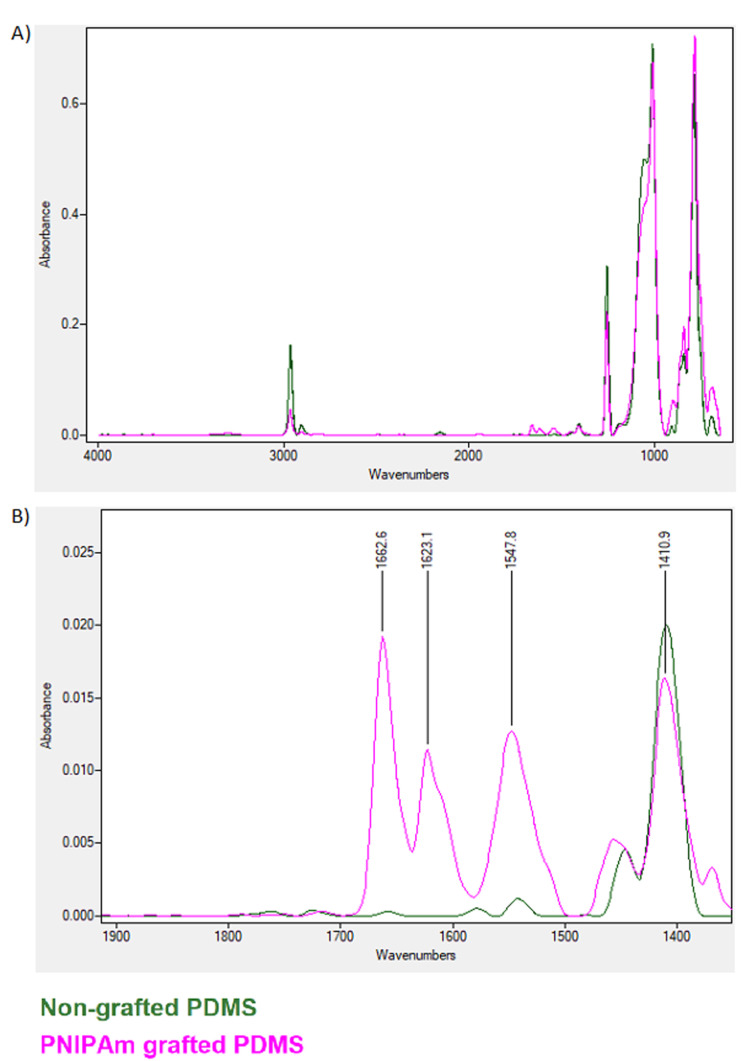
ATR FTIR spectrum for grafted versus non-grafted PDMS. Results indicated the successful NIPAAm grafting and amide peak formation between 1500-1700 wavenumbers.

ATR-FTIR analysis of the PNIPAAm-grafted and non-grafted cell-imprinted PDMS surfaces was done and compared to the pure NIPAAm monomer (Figure 5). The overlap of absorption bands in PNIPAAm-grafted and non-grafted PDMS is almost the same except in the region between 1500 and 1700 (Figure 5a). While amide II bands were not observed in non-grafted PDMS, indicating the successful integration of PNIPAAm to the PNIPAAm-grafted PDMS surface. These data indicate partial contributions of N–H bending and C–H stretching of the amide group in the grafted PNIPAAm polymer.

### Transdifferentiation of the AT-MSCs into KLCs

**Figure 6:**
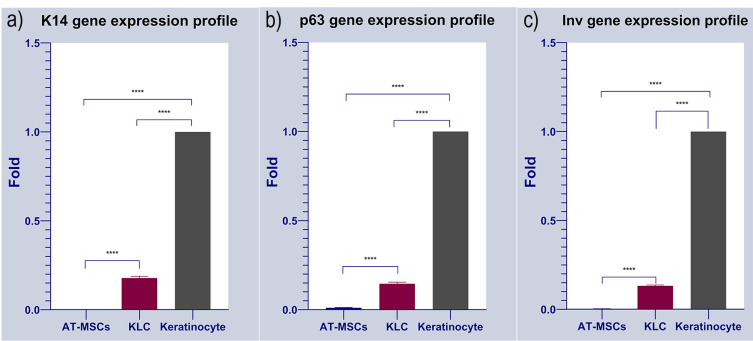
Gene expression profile of cultured AT-MSCs on the cell-imprinted thermoresponsive PDMS substrate have been evaluated by keratinocyte markers such as Cytokeratin 14 (K14), Involucrin (Inv) and p63. Keratinocyte and AT-MSCs were used as positive and negative control, respectively. It can be observed that the expression of Cytokeratin 14 (K14), Involucrin (Inv) and p63 were increased in transdifferentiated KLCs cultured on the keratinocyte imprinted substrate. As shown here, there exist no expression data in AT-MSCs as the negative control of the study. Keratinocytes were normalized to 1 since these cells possess these markers. Data shows the positive expression of the all selected markers in KLC after transdifferentiation compared to AT-MSCs, Cytokeratin-14, 0.178323632 vs. 0.00028413 (****P < 0.0001); involucrin, 0.003294605 vs 0.132567826 (****P < 0.0001) and p63, 0.010602757 vs 0.145643358 (****P < 0.0001); respectively) (****P < 0.0001; n = 3 independent experiments).

The expression of multiple keratinocyte-specific genes like K14, Inv, and p63 was monitored in AT-MSCs cultured on substrates’ surface. Data showed a statistically significant difference in the expression of selected genes between groups (*P* < 0.05; Figure 6). As expected, AT-MSCs did not express keratinocyte-associated markers K14, Inv, and p63. Of note, in the positive control, KLCs, transcription of all three genes were evident. Compared to the AT-MSCs group, we found a statistically significant difference in KLCs (Figure 6). These data showed that AT-MSCs are devoid of KLC-associated factors. Based on our data, we found that 14-day culture of AT-MSCs on PDMS substrate increased the expression of K14, Inv, and p63 compared to the AT-MSCs (*P* < 0.05). To the AT-MSCs at the mRNA level, the fold change results was (Cytokeratin-14, 0.178323632 vs. 0.00028413 (****P < 0.0001); involucrin, 0.003294605 vs 0.132567826 (****P < 0.0001) and p63, 0.010602757 vs 0.145643358 (****P < 0.0001); respectively) (****P < 0.0001; n = 3 independent experiments). To conclude, KLCs demonstrated gene expression profiles of the keratinocyte-specific markers similar to those of the keratinocytes. These results indicated that PDMS induces KLC-differentiation of AT-MSCs possible *in vitro*.

### Histological analysis of the detached KLCs sheet

**Figure 7:**
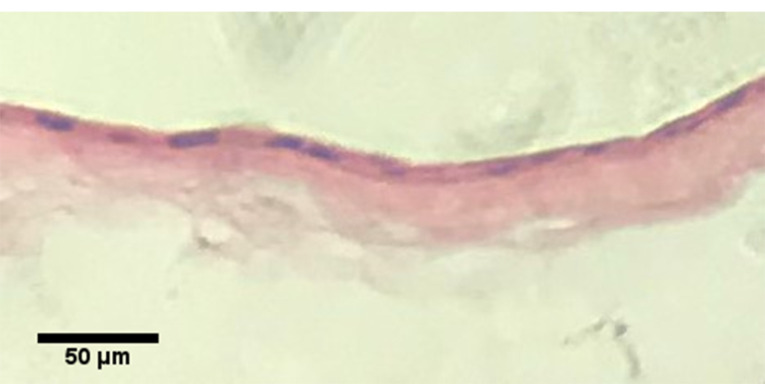
Hematoxylin & Eosin staining of the KLC sheet.

The formation of multilayer KLCs was analyzed using H&E staining (Figure 7). The data exhibited the overlapping of KLCs in the thermo-sensitive scaffold, showing the applicability of PDMS substrate in the regeneration of skin diseases.

Skin regeneration is an important field of regenerative medicine. Since stem cells can trans-differentiate into functional cells, thereby they play a very important role in the regeneration of different organs.^3,5,41,42^ Among all the stem cell types, AT-MSCs can be easily harvested from fat tissue samples. Like bone marrow MSCs, AT-MSCs possess plasticity, high proliferation capacity, paracrine activity, and immunomodulatory properties. These features make AT-MSCs more advantageous to other adult stem cells.^43^ AT-MSCs, not only, can differentiate into mesenchyme germ layer but also commit into other different germ layers, a phenomenon known as “Trans-differentiation’’. However, for the successful orientation of these cells toward target cell lineages, the existence of special environmental topographies is vital like stimulatory factors and suitable scaffolds.^43^ It is known that stem cells are highly sensitive to their environmental chemistry, stiffness, and more importantly to the topography of their culture substrate.^22^ Different studies have been conducted using the various extracellular matrix (ECM) like topographies in micro and nano-sized triangle, round and multigonal formats. Based on data, all these features can affect cell attachment, spreading, cytoskeletal architecture, nuclear shape, and orientation into specialized cell types.^10,13,22-24^

Cell-imprinted PDMS substrates is a promising material for various applications like microfluidic systems, molding, and developing desired substrates. Moreover, PDMS is a transparent and biocompatible material and has great advantages for biomedical applications.^44^

PNIPAAm is a well-known and widely used smart polymer that shows a temperature-responsive manner and hydrophobic/hydrophilic phase transition in an aqueous solution. PNIPAAm exhibits both upper-critical solution temperature (UCST) or lower-critical solution temperature (LCST, higher than body temperature) phase behaviors.^45^ Since the LCST of PNIPAAm is near the body temperature (at 32°C), it is a good candidate for various biomedical applications like drug/gene delivery systems.^46-48^ First, Okano et al. used PNIPAAm in cell culture to control the cell attachment/detachment capacity by changes in environment temperature. This capacity will help scientists to create a monolayer of cells having the intact ECM (which is essential for the efficient cell adhesion, differentiation, and their tissue-like function) on their basal layer called “cell sheet”.^49^

Here, we performed a cell-imprinted substrate to control the differentiation of AT-MSCs toward KLCs. For this purpose, keratinocyte cell shape topography was induced on the PDMS silicone and grafted to thermoresponsive polymer PNIPAAm. Ultrastructural analysis revealed 20-µm grooves after the culture of keratinocytes on the PDMS substrate. These data showed the efficiency of the current protocol in the induction of cell-imprinted topography on the thermo-sensitive substrate. Further analysis by ATR-FTIR showed efficient integration of PDMS to PNIPAAm. To be specific, the overlap of absorption bands in PNIPAAm-grafted and non-grafted PDMS is almost the same except in the region between 1500 and 1700. The region around 1600 and 1500 are indicators of the success amid bonds which represents the C = O and C-N respectively. We also showed that the expression of genes such as K14, Inv, and p63 increased after 14-day culture on the scaffold surface with certain topographical features. P63 is a homolog of the p53 transcription factor which represents the epithelial development and proliferation. This marker is known for distinguishing the keratinocyte stem cells of the basal layer from the more specialized transient amplifying progenitors. This factor is also the indicator of the epidermal differentiation and basement membrane formation and thus can be a representative for the keratinocyte progenitor cells.^50^ Besides p63, the basal layer keratinocyte stem cells contain keratin bundles such as cytokeratin 14 (K14) and cytokeratin 5 (K5).^50^ Other structural proteins such as involucrin are also synthesized during the very first steps of the keratinocyte differentiation.^51^

Finally, after culturing the AT-MSCs on PNIPAAm grafted substrate, as shown in Figure 6, qPCR analysis showed that, KLC started to all keratinocyte specific markes (K14, Involucrin and p63) comparing to our negative control (AT-MSCs cultured on non-imprinted substrate). The fold change results was (cytokeratin-14, 0.178323632 vs. 0.00028413 (****P < 0.0001); involucrin, 0.003294605 vs 0.132567826 (****P < 0.0001) and p63, 0.010602757 vs 0.145643358 (****P < 0.0001); respectively) (****P < 0.0001; n = 3 independent experiments). Finally, desired cell sheet was removed from the substrate 20-30 minutes after replacing the medium with chilled one and analyzed with H&E staining. As shown in Figure 7, the uniform cell sheet with blue cell nucleus and red cytoplasm is obvious.

## Conclusion

In this study, the desired keratinocyte cell-imprinted substrate was successfully developed. The culture of AT-MSCs induced KLC like phenotype after 14 days. Along with morphological adaptation, the expression of K14, Inv, and p63 increased in AT-MSCs. In conclusion, the results of this study confirmed that keratinocyte cell-imprinted substrate could dictate KLC like phenotype in AT-MSCs cultured on the thermoresponsive cell-imprinted substrate.

## Acknowledgments

This study has been extracted from the thesis registered in the Stem Cell Research Center, Tabriz University of Medical Sciences (No. IR.TBZMED.REC.1395.776). We thank our colleagues who provided insight and expertise that greatly assisted the research. This study was funded by the stem cell research center of the Tabriz University of Medical Sciences, Tabriz, Iran.

## Ethical Issues

The ethics committee of the Tabriz University of Medical Sciences has approved this study. All human foreskin tissue samples were obtained following written informed consent from Alzahra hospital, Tabriz, Iran.

## Conflict of Interest

The authors declare no conflict of interest.
